# Multiscale CNNs for Brain Tumor Segmentation and Diagnosis

**DOI:** 10.1155/2016/8356294

**Published:** 2016-03-16

**Authors:** Liya Zhao, Kebin Jia

**Affiliations:** Multimedia Information Processing Group, College of Electronic Information & Control Engineering, Beijing University of Technology, Beijing, China

## Abstract

Early brain tumor detection and diagnosis are critical to clinics. Thus segmentation of focused tumor area needs to be accurate, efficient, and robust. In this paper, we propose an automatic brain tumor segmentation method based on Convolutional Neural Networks (CNNs). Traditional CNNs focus only on local features and ignore global region features, which are both important for pixel classification and recognition. Besides, brain tumor can appear in any place of the brain and be any size and shape in patients. We design a three-stream framework named as multiscale CNNs which could automatically detect the optimum top-three scales of the image sizes and combine information from different scales of the regions around that pixel. Datasets provided by Multimodal Brain Tumor Image Segmentation Benchmark (BRATS) organized by MICCAI 2013 are utilized for both training and testing. The designed multiscale CNNs framework also combines multimodal features from T1, T1-enhanced, T2, and FLAIR MRI images. By comparison with traditional CNNs and the best two methods in BRATS 2012 and 2013, our framework shows advances in brain tumor segmentation accuracy and robustness.

## 1. Introduction

Brain tumor is an uncontrolled growth of solid mass formed by undesired cells found in different parts of the brain. It can be divided into malignant tumor and benign tumor. Malignant tumors contain primary tumors and metastatic tumors. Gliomas are the most common brain tumors in adults, which start from glial cells and infiltrate the surrounding tissues [1]. Patient with low grade gliomas can expect life extension of several years while patient with high grade can expect at most 2 years [2]. Meanwhile, the number of patients diagnosed as brain cancer is growing fast year by year, with estimation of 23,000 new cases only in the United States in 2015 [3].

Surgery, radiation, and chemotherapy are mainly the common treatments for brain tumors. The latter two aim to slow the growth of tumors, while the former one tries to resect and cure tumors. Thus early diagnosis and discrimination of brain tumor become critical. At the same time, accurate location and segmentation of brain tumor are essential for treatment planning.

Among the variety of imaging modalities, Magnetic Resonance Imaging (MRI) shows most details of brain and is the most common test for diagnosis of brain tumors [4, 5]. MRI contains T1-weighted MRI (T1w), T1-weighted MRI with contrast enhancement (T1wc), T2-weighted MRI (T2w), Proton Density-Weighted MRI (PDw), Fluid-Attenuated Inversion Recovery (FLAIR), and so forth. Unlike Computed Tomography (CT) image, MRI images from different types of machines have different gray scale values. Gliomas can appear in any location of the brain in any size and shape. Also gliomas are infiltrative tumors and are difficult to distinguish from healthy tissues. As a result, different information provided by different MRI modalities should be combined to settle the difficulties mentioned above.

Although decades of efforts have been made to look for efficient methods for brain tumor segmentation, no perfect algorithm has been found. Besides, most are based on conventional machine learning methods or segmentation methods for other structures [6]. These methods either use hand-designed specific features or produce bad segmentation results when tumor surroundings are diffused and poorly contrasted. Methods based on hand-designed specific features need to compute a large number of features and exploit generally edge-related information while not adapting the domain of brain tumors. Besides, traditional feature produced through image gradients, Gabor filters, Histogram of Oriented Gradients (HoG), or Wavelets shows bad performance especially when boundaries between tumors and healthy tissues are fuzzy. As a result, designing task-adapted [7] and robust feature representations is essential for complicated brain tumor segmentation. Recently, Convolutional Neural Networks (CNNs) as supervised learning methods [8] have shown advantages at learning hierarchy of complex features from in-domain data automatically [7] and achieved promising results in facial [9] and MINST database recognition [10], mitosis detection [11], and so forth. Besides, CNNs have also shown successful application to segmentation problems [12, 13], while they are not often used in brain tumor segmentation tasks [14, 15]. However, traditional standard CNNs only focus on local textual features. As a result, some important global features are lost inevitably. As both local and global features play an important role in image recognition tasks [16, 17], we propose a specific CNNs architecture for brain tumor segmentation combining all these features. In this architecture, multiscale concept is introduced to our previous designed traditional single-scale CNNs [18]. By combining multiscale CNNs, both local and global features are extracted. The pixel classification is predicted by integrating information learned from all CNNs. Besides, multimodality MRI images are trained at the same time for utilizing complementary information. Experiments show promising tumor segmentation results through multiscale CNNs.

## 2. Our Multiscale Convolutional Neural Networks

In order to cut computation time caused by 3D CNNs, we utilize 2D CNNs from axial view, respectively. As MRI image lacks information from *z* axial, *x*-*y* slice patches are used and, through this way, computation time is greatly reduced. For each 2D axial image, each pixel combines information from different image modalities, including T1, T2, T1c, and FLAIR. Just like traditional CNNs [19, 20], our multiscale CNNs model takes the class of the central input patch as prediction result. Thus the input patch of the network is several 2D patches from four modalities.

### 2.1. Traditional CNNs

**Figure 1:**
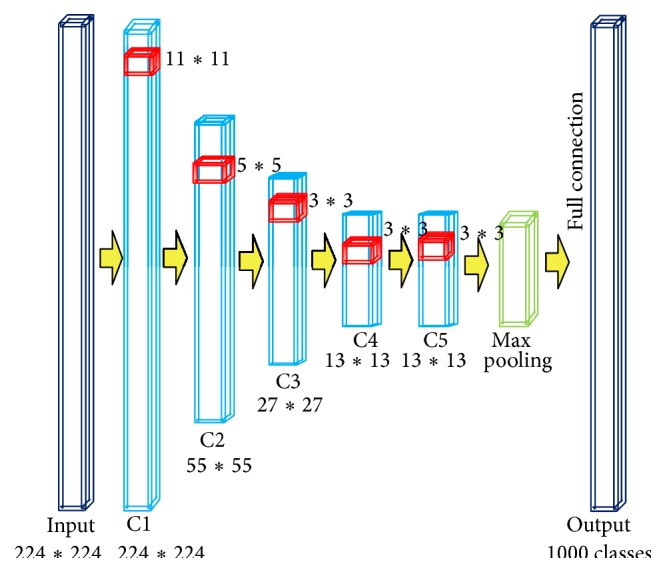
The architecture of traditional ImageNet CNNs.

**Figure 2:**
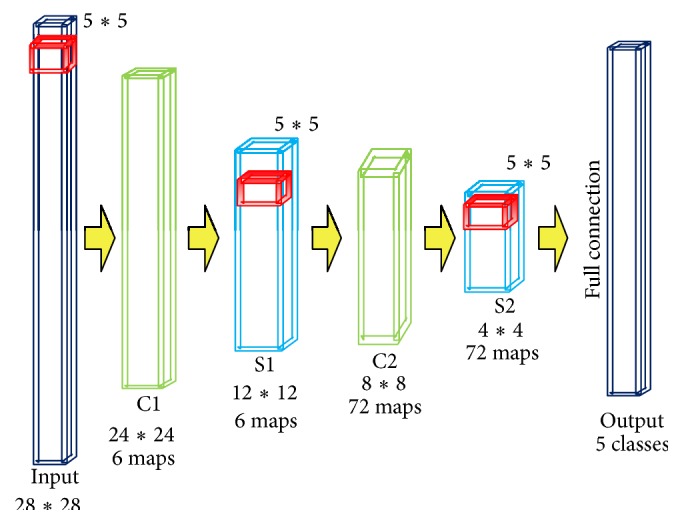
The architecture of traditional MNIST CNNs.

In order to clearly show structures of CNNs, Figures 1 and 2 are two classical examples of traditional CNNs for the famous ImageNet [21] and MINST [10] datasets. After the input layer, as shown in the figures, CNNs are composed of two parts: the feature extraction part and the full connection of classification part. The feature extraction part consists of pairs of convolution layer and down sample layer, through which hierarchy of features is extracted. Output of each layer acts as input of the subsequent layer pair. This output is feature map of that layer. As a result, trivial parts of images are cut down and main description of input image patches is left, respectively.

According to different data structures, the CNNs utilize different sizes of patches as inputs (red in Figures 1 and 2; 224*∗*224 for ImageNet and 28*∗*28 for MNIST, which is static for one specific dataset). Reasonably, for one specific type of data, only one network structure is appropriate (as ImageNet CNNs and MINST CNNs). From the sample in Figure 1, we can conclude that traditional CNNs run well on the key features detection and recognition. The images in ImageNet are usually nature images to which the local features (or texture features) are more important than the global features (or the structure features). Traditional CNNs perform pretty well for training classification of one specific problem. But it may crash for using only one network structure to handle such variable region problems. Brain tumor can be of any size and shape in different patients, and even different slices of one patient are not the same size. Besides, as traditional CNNs focus too much on local features, the trained network is not adaptive for different input brain tumor images. For the tumor images in which the features are usually fuzzy, both local and global features are very important. As a result, the accuracy for recognizing tumor through traditional CNNs would be decreased heavily.

To solve this kind of problem, we propose a multiscale CNNs framework which could detect both local and global features at the same time. The multiscale CNNs, which combine both local and global features, could be adaptive to different resources of input images, including different resources of brain tumor and other soft tissues.

### 2.2. The Architecture of Our Multiscale CNNs Model

#### 2.2.1. Automatic Selection of Proper Image Patch Size in Multiscale CNNs Model

**Figure 3:**
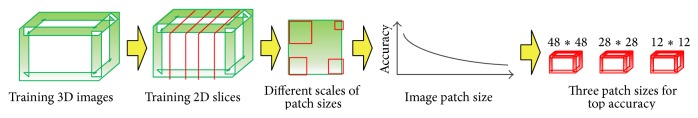
The workflow of automatic selection of proper image patch size.

**Figure 4:**
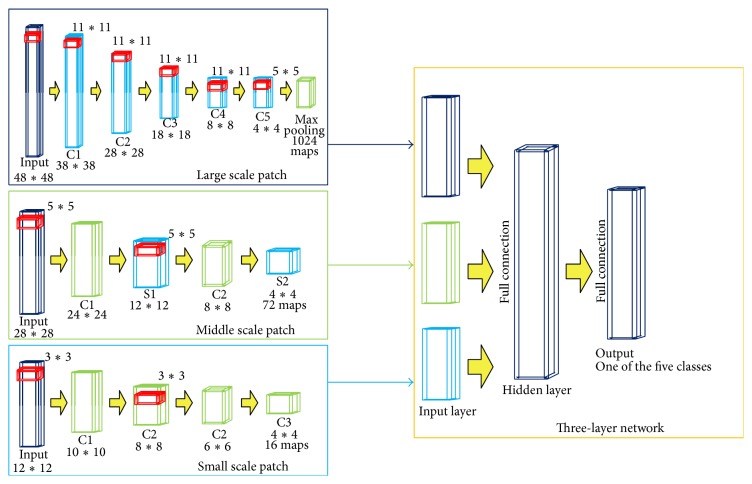
The architecture of multiscale three-layer neural network.

From discussion in Section 2.1, selection of proper input image patch size is a critical part for extracting features. Choosing proper size, especially close to that of brain tumor, can improve classification accuracy of CNNs. This is one point we seriously emphasize through experiments. The workflow of our automatic selection of proper image patch size is shown in Figure 3. Traditional procedure in classification includes training and testing parts. Here we make a prior procedure before the training part. As Figure 3 shows, 1% of training 2D slices data is randomly selected and different scales of image patches are extracted in order to get the top-3 image patch sizes. This three-channel data is the source of multiscale CNNs in Figure 4. In this paper, from the prior procedure, three scales of patches 48*∗*48, 28*∗*28, and 12*∗*12 produce the top-three accuracy results and are selected as the source of the multiscale CNNs. When datasets change, by the procedure of prior method, different scales of image path sizes would be selected for the best classification result.

#### 2.2.2. Our Multiscale CNNs Architecture

The overall structure of multiscale CNNs is shown in Figure 4. This network is fit for a wide range size of brain tumor. We propose three-pathway CNNs architecture. This architecture is constructed with three-pathway streams: one way with small input image size 12*∗*12, one with middle input image size 28*∗*28, and last one with large size 48*∗*48. Under this architecture, the prediction of each pixel's classification is made through combination of different scales of features: both the local around that pixel and the global region.

Taking 48*∗*48 block size, for example, we design seven-layer architecture, including one input layer, five convolutional layers (noted as C1, C2, C3, C4, and C5), and one max pooling layer. In this architecture, convolutional layers act as building blocks of CNNs. These convolutional layers are stacked on top of each other to form a hierarchy of features. Filter kernel size of each convolutional layer is set to 11*∗*11, 11*∗*11, 11*∗*11, 11*∗*11, and 5*∗*5. The first convolutional layer C1 takes different modalities of MRI image patches as inputs and produces 12 feature maps of size 38*∗*38. Then the output of C1 acts as inputs of C2. Feature map is computed as

(1) $$\begin{array}{c} M_{s}=b_{s}+\underset{c}{\sum }W_{sc}*X_{c}, \end{array}$$

where *X*_*c*_ is the *c*th input channel (here one modality as per input channel), *W*_*sc*_ is the kernel in that channel, *∗* is the convolution operation, and *b*_*s*_ is the bias term. After five convolutional layers, max pooling operation takes place. This is to take the maximum feature value over subwindows within each feature map, shrinking the size of the corresponding feature map. This subsampling produce introduces the property of invariance to local translations. Weights of three pathways are learnt separately and then are combined together for a three-pathway network. Success of our proposed multiscale CNNs owes to these data-driven and task-specific dense feature extractors.

After all these different task-specific hierarchies of features extracting procedures, all outputs of three pathways are combined as input of a full connection layer. This full connection layer is for final classification of the central pixel of that input image patch. In this layer, learnt hierarchical features of three pathways are arranged in one dimension and utilized together for patch classification.

The detailed procedure is as follows: for the first channel (in which the image patch size is 48*∗*48) after max pooling procedure, produced feature number and size are 1024 and 4*∗*1; for the second channel (in which the image patch size is 28*∗*28), the output number and size of feature are 72 and 4*∗*4, respectively; for the third channel (in which image patch size is 12*∗*12), the output feature size is 4*∗*4 and feature number is 16. In order to combine these three kinds of outputs which are different from each other in both feature size and number, a new one-dimensional vector is made which contains all the features in three channels, and the vector size is 5504 (the same with the sum of three kinds of features). This vector is the description of the tumor image and plays the role in the tumor classification in next three-layer module.

Detailed experiment performance analysis is described in Section 3.

## 3. Experiment Results

In this section, in order to show the effectiveness and robustness of our multiscale CNNs architecture, various kinds of experiments are carried out. Firstly, it is important to determine the proper image patch sizes for inputs in the prior procedure before training. This operation is to find the most proper patch size for CNNs training. Experiments are carried out to test performance of traditional CNNs with only one pathway. In this section, performances of CNNs with different layers and iteration times are also tested. Then any two traditional CNNs tested in former section are combined as two-pathway CNNs and comparisons are made among different number pathways of CNNs. Finally, experiments are carried out to show the high segmentation accuracy and efficiency of our designed three-pathway CNNs architecture. Comparison is made among published state-of-the-art methods in BRATS [1].

Dice ratio is commonly utilized to validate algorithm segmentation result. As defined in (2), dice ratio is to show similarity between manual standard and our automatic algorithm segmentation result:

(2) $$\begin{array}{c} DR\left(\;A,B\;\right)=\frac{2\left|A\cap B\right|}{\left|A\right|+\left|B\right|}. \end{array}$$

### 3.1. Data Description

**Figure 5:**
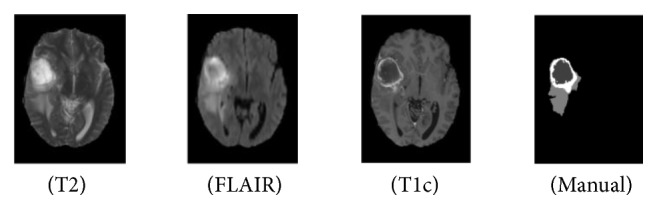
The three types of data and the manually generated results.

The experiments are performed over datasets provided by benchmark BRATS 2013 [1]. The datasets include T1, T1c, T2, and FLAIR images. The training data includes 30 patient datasets and 50 synthetic datasets. All these datasets have been manually annotated with tumor labels (necrosis, edema, nonenhancing tumor, and enhancing tumor denoted as 1, 2, 3, and 4, while 0 stands for normal issue).  Figure 5 shows an example of tumor structures in different image modalities (T1, FLAIR, T1c, and manual standard). For tumor detection, T1 shows brighter borders of the tumors, while T2 shows brighter tumor regions. FLAIR is effective for separating edema region from CSF. In this paper, the four image modalities are combined to utilize complementary information in CNNs training.

### 3.2. Proper Image Patch Size and Layer Number for Traditional CNNs

**Figure 6:**
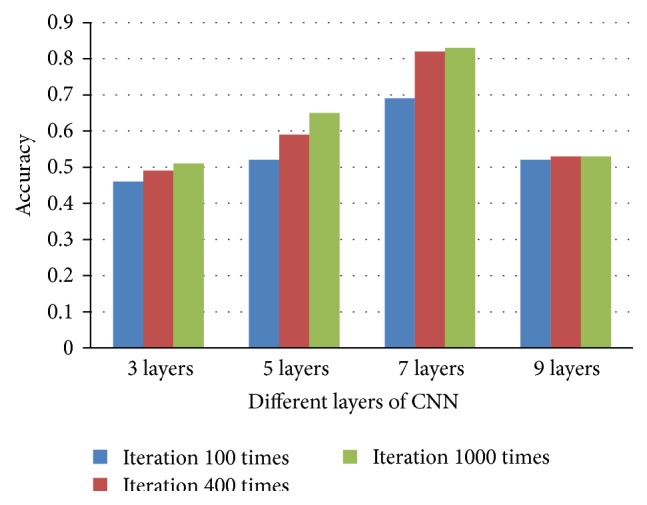
The comparison of different layers of traditional CNNs.

As traditional CNNs have only one network structure, it is fit for certain fixed size of input image patch. We get the top-three image patch sizes in the prior procedure before training (as shown in Figure 3).  Figure 6 shows the mean brain tumor segmentation results on five datasets. We designed various one-pathway CNNs architecture including 3, 5, 7, and 9 layers. At the beginning, brain tumor segmentation accuracy increases with the increase of layer number. Structure with 7 layers for input patch size of 48*∗*48 reaches top accuracy. But accuracy falls greatly in 9-layer CNNs.

For each iteration time, segmentation result is refined and rectified. As a result, accuracy also goes higher with the increase of iteration times (in Figure 6). Through experiment, it can be concluded that iteration time of 1000 is a most proper choice.

**Figure 7:**
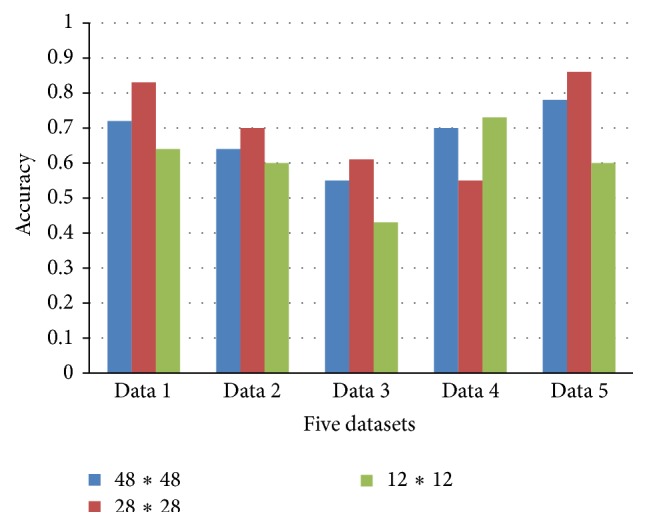
The comparison of different patch size of traditional CNNs.

Figure 7 illustrates the different influences of top-three input image patch sizes on brain tumor segmentation result of traditional one-pathway CNNs. Statistics is made on test dataset from 1 to 5. 48*∗*48, 28*∗*28, and 12*∗*12 are the three most proper types of patch size in conclusion. It can be drawn that, among these three types, 28*∗*28 is the best choice in most datasets (according to data 1, 2, 3, and 5 except 4). 28*∗*28 performs not so well as the other two types in dataset 4, which is probably because of the complex shapes of tumors in different slices and locations. But its accuracy is still higher than that of 48*∗*48 and 12*∗*12 patch size in dataset 3. As a whole, these three types of patch size grasp different scales of features and information from brain tumor images, which are all important in tumor segmentation and recognition.

Experiment in this section shows that the patch size and the layer of the designed CNNs architecture all play important roles in the accuracy of tumor classification. As Figures 6 and 7 show, the three proper patch sizes (i.e., 48*∗*48, 28*∗*28, and 12*∗*12) should be the main patch sizes for our multiscale CNNs network, each as one pathway.

### 3.3. Comparison between Combination of Different Scale CNNs

**Figure 8:**
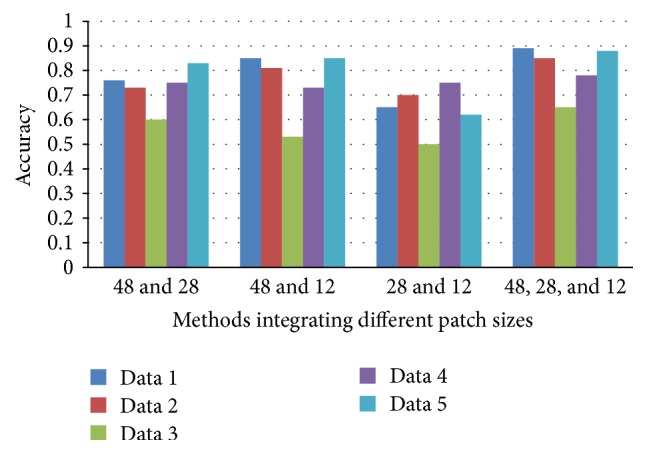
The accuracy results of methods integrating different patch sizes.

Combination of any two of the top-three proper patch sizes, that is, forming two-pathway CNNs, is tested in this section. And finally each of the patch size CNNs acts as one pathway of our multiscale CNNs. Comparison is also made among them. From Figure 8, it is clear that through joint training of any two-scale CNNs, tumor classification accuracy is improved compared with single path CNNs, but in different improvement extent. As a whole, joint training containing 48*∗*48 patch size performs better than the other two patch sizes (28*∗*28 and 12*∗*12). This is because 48*∗*48 patch size provides global scale information for CNNs feature learning. Pity that 28*∗*28 and 12*∗*12 joint training even reduces accuracy compared with 28*∗*28 single pathway CNNs, but higher than that of 12*∗*12 CNNs. This is probably because of the lack of global features in 12*∗*12 path pathway. It is obvious that through combining both global and local features (48*∗*48, 28*∗*28, and 12*∗*12 in this paper), accuracy is improved and reaches nearly 0.9 for some dataset in Figure 8, which illustrates the advantages of our proposed multiscale CNNs model.

### 3.4. Comparison with Other Methods

**Figure 9:**
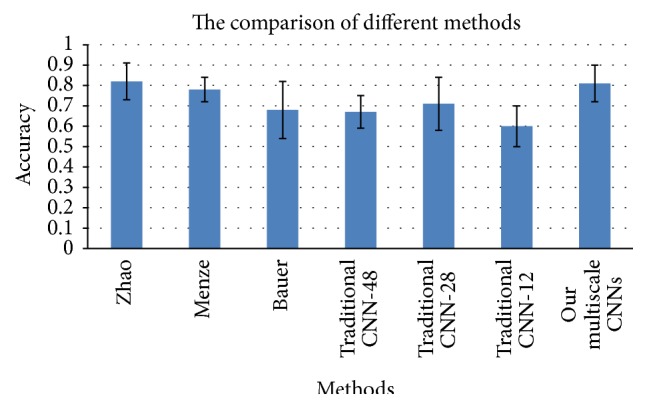
The comparison with other methods.

Figure 9 illustrates comparison with other methods in BRATS challenge [1]. Paper [1] published in 2014 gives comprehensive and formal results of methods that participated in BRATS 2012 and 2013. We select the best two performing algorithms [1, 18] for comparison. Top edge of the blue rectangle stands for the mean accuracy of each method, while whisker across the average value indicates variance of each method.

Without exception, average accuracy results of traditional one-pathway CNNs of 48*∗*48, 28*∗*28, and 12*∗*12 all fall behind the top two methods. However, performance of traditional CNNs with path size of 28*∗*28 is still better than Bauer 2012, which illustrates that patch size 28*∗*28 is a most proper choice. While mean score of our multiscale CNNs is lower than the best method (0.81 versus 0.82), our method is almost as stable as the best method (variance 0.099 versus 0.095). Compared with second best method Menze, our method is more accurate (0.81 versus 0.78) but not as stable as it (0.099 versus 0.06). This is probably because of the lack of specific preprocessing step before CNNs training.

## 4. Conclusion

In this paper, we propose an automatic brain tumor segmentation method based on CNNs. Traditional one-pathway CNNs only support fixed size of input image patches and relay on local features. Besides, brain tumor can be of any size and shape and locate in any part of the brain. As a result, traditional CNNs are not adaptive to accurate segmentation and classification of brain tumor. To solve all the above problems, we present a multiscale CNNs model, through which not only local and global features are learnt, but also complementary information from various MRI image modality (including T1, T1c, T2, and FLAIR) is combined. Through various experiments, our three-pathway CNNs show their advantages and improvements compared with traditional CNNs and other state-of-the-art methods.

In the future, we shall explore and design other CNNs architectures to further utilize its self-learning property and improve segmentation accuracy by extracting richer boundary information and discriminating fuzzy points.
